# Metropolitan Hospital Sunday Fund

**Published:** 1897-11-06

**Authors:** 


### The Institutional Workshop.

### METROPOLITAN HOSPITAL SUNDAY FUND.

### THE OUT-PATIENT QUESTION.

A meeting of the Council of the Metropolitan Hospital Sunday Fund was held at the Mansion House on Wednesday, 27th ult., to receive a special report from the Distribution Committee with reference to out-patients. Sir Sydney H. Waterlow, Bart., in the absence of the Lord Mayor, took the chair, and those present included: The Earl of Stamford, Sir W. H. Broadbent, Bart., Sir Henry Burdett, Sir Edmund Hay Currie, the Hon. Sydney Holland, Messrs. Thomas Bryant, A. G. Sandeman, J. H. Hale, F. C. Carr-Gomm, H. Brooks, W. R. Tidd, George Drummond, A. Willett, and Thomas Wakley, Dr. Glover, Dr. Hare, Captain Cundy, the Revs. Canon Graham, Canon Rhodes Bristowe, Prebendary Kitto, R. R. Connell, C. H. Grundy, H. H. Harward, Dr. Rigg, W. H. Barlow, M. R. Neligan, R. S. Hassard, and others.

The Chairman, after referring to the great loss which the nation had sustained by the sudden death of the Duchess of Teck, said that that meeting had been called because the Distribution Committee had felt that the subject of the meeting—the out-patient question—ought to be discussed before the ordinary meeting of the constituents of the Fund in December. The subject had been under the consideration of the members of the Distribution Committee for four or five years past. As far back as 1892 they had had before them evidence on the subject, and since then, year after year, a great deal of information had come under their notice. Having, therefore, carefully considered all the information at their disposal, they had come to the conclusions set forth in the report they now presented, which was as follows:

"The Committee of Distribution having carefully reviewed all the evidence received by them during the last four or five years in reference to the out-patient departments of the several hospitals, and having recently obtained further information on this important subject, are of opinion that, where no person has been already appointed, efforts should be made to induce the governing bodies of hospitals to appoint efficient persons to make inquiries respecting patients attending out-patient departments, with the view of preventing any abuse, and that the Council of the Hospital Sunday Fund should exercise the greatest caution before interfering with the internal management of hospitals."

The committee had had many difficulties to contend with, and the chief amongst them was the very great diversity of opinion on the question which was to be found amongst the very people to whom the committee had to look for guidance. In proof of this the speaker contrasted several of the opinions given at the recent meeting of the Hospital Reform Association at St. Martin's Town Hall. A great point, Sir Sydney Waterlow contended, had been made of the number of people who attended the out-patient departments, but it must be remembered that, although the figures quoted might correctly give the number of cases, yet the number of people who attended would be far less, as from his own experience of the out-patient department at St. Bartholomew's Hospital he knew that many of the out-patients who attended for trivial complaints came back again a month or two months after they were cured, perhaps four or five times in the year, for some other ailment. The first remedy, he believed, for the present evil was the appointment of an efficient enquiry officer. He agreed with a speaker at the meeting he had already referred to, that progress in the matter must be slow, because there would be great difficulty in obtaining the full concurrence and assistance of the staffs of the large hospitals, where the largest number of out-patients attended. He moved that the report of the Distribution Committee be approved and adopted.

Sir Edmund Hay Currie, in seconding, said he thought that, although this report was but a small thing to give them after five years' work, yet still he welcomed it, as it was a step in the right direction. Anyone who thought of the enormous—the perfectly marvellous—improvement which had taken place during the last thirty or forty years inside our hospitals, must be a little horrified to see that no improvement had taken place in the out-patient departments. Forty or fifty years ago the hospitals of London had to treat the destitute poor, but since the passing of the Gathorne Hardy Act in 1840, the great improvement in the Poor Law system had taken the destitute poor away from the hospitals, and the persons who now used them were the wage-earners, people who earned from 25s. to 40s. a week. It was, he thought, a hardship to those persons that they should not be allowed to pay for their treatment; they did not want charity, but they wanted to get the very best advice at a small cost. Of course, the ideal system would be to have provident dispensaries all over London working up to the hospitals, which should stand in the same relation to the dispensaries as the consultant did to the general practitioner. It was, however, almost impossible to start a provident dispensary in the neighbourhood of a large hospital, like, say, the London Hospital.

Dr. Glover suggested that the report should be entered on the minutes, and that then he would propose the following resolution, which would carry the matter a step further. He did not wish to propose it as an amendment to the Chairman's resolution, but rather as a corollary. His resolution was:—

"That the Council of the Hospital Sunday Fund, having before them the yearly statistics of the metropolitan hospitals and the report of the Distribution Committee on the out-patient department of hospitals, is of opinion that the large and increasing number of out-patients is a matter calling for the anxious attention of the managers of hospitals; and that this Council, while disclaiming any wish to interfere with the internal management of hospitals, will look upon the appointment of efficient inquiry officers, and other means of curtailment, as points of merit in administration."

The Chairman suggested that the report should first be received, and then he was prepared to move a resolution recommending the constituents of the Fund to add a law making it a merit where the committee felt that there was an efficient, careful, and earnest attempt to prevent abuse in the out-patient department.

Sir Henry Burdett said he was most anxious that that report of the Distribution Committee should be adopted, as he thought it embodied the kernel of the means by which the only effective remedies for out-patient abuse could be applied. He was confident that if that system of inquiry was applied at the entrance to the out-patient department, and if the Fund took the trouble to see that it was conducted by trained and efficient officers, who had the desire to do the work in a kindly, friendly, and efficient spirit, they would very soon be able to erect upon that foundation other changes which collectively would make out-patient abuse impossible.

Dr. Glover agreed to this.

The Rev. C. H. Grundy moved an amendment to the effect that investigation as to the suitability of an out-patient for relief should be the duty of the responsible persons in the locality in which the applicant resided, and that each application for hospital relief should be signed by some minister of religion or by some officer of the Charity Organisation Society, or by two ratepayers who knew the applicant. All these agencies were already present in the districts, and a great saving in expense would result to the hospitals. There was no seconder, however, and the amendment was dropped.

The Hon. Sydney Holland did not think that those who were working in hospitals every day ought to admit that there was the great abuse which had been made so much of. He therefore, as the chairman of three hospitals, protested against the assumption that there was this great abuse of the out-patient department. As a result of inquiries made by the Charity Organisation Society at the Royal Free Hospital, it was found that, of the out-patients who attended there during a year, 70 per cent. were proper cases and 30 per cent. ought to have been referred to the Poor Law. That, he thought, was an answer to Sir Edmund Hay Currie, who said that the destitute poor no longer attended the hospitals. The destitute poor did attend the hospitals because they were dissatisfied with the relief they got from the Poor Law, and if this was to be prevented the Poor Law system must be strengthened. Sir Edmund Hay Currie had also said that hospitals like the London Hospital prevented the existence of provident dispensaries. A curious answer to that statement was the fact that the most successful provident dispensary in London was exactly opposite the doors of the London Hospital. Another interesting fact was that, so far from this provident dispensary being delighted with the proposal to charge the out-patients at the London Hospital for medicine, the chairman had in a recent speech complained that the London Hospital was about to become a rival to the dispensary, so that apparently it would appear that a hospital where payment by patients was proposed was considered to be more detrimental to providence amongst the poor than one where the treatment was given free.

The adoption of the report was agreed to unanimously.

The Chairman moved the following resolution :—

"That this Council recommend that at the next meeting of the constituents in December the following clause be added to the laws of the constitution: 'That where any hospital having an out-patient department has appointed a special and efficient officer for detecting any abuses in that department, it shall be regarded by the Distribution Committee as an important factor in determining merits.'"

Sir W. H. Broadbent, Bart., seconded. He thought the step taken in that resolution was a very important one, more especially as it affected the smaller general and the special hospitals, which actually competed for out-patients. Those out-patients who went to hospitals to which no medical school was attached in no way served the cause of medical education. They might supply a certain amount of experience to physicians and surgeons, and they certainly supplied a considerable amount of advertisement for the hospital.

Dr. Glover, in supporting the resolution, said that however much the numbers of the out-patients might be minified by people, it must be admitted that they were enormous. Whereas in 1895 the number of out-patients treated in London was 1,098,224, in 1896 the number was 1,141,444, or an increase in one year of 43,000. Taking a few individual hospitals, the increased number of out-patients in 1896 when compared with 1895 was very large. For instance, at the Great Northern Central Hospital there had been an increase of over 3,000; at Guy's one of nearly 2,000; at the London, 3,385; at the Metropolitan, 5,425; at the Royal Free, 5,032; at the Middlesex, 1,562; and at the Westminster, 4,2[illegible]7.

After some further discussion, the resolution was carried unanimously.

Hospital Chaplains.

Sir Henry Burdett said that at a conference of clergy and ministers held some little time back the question of the religious disabilities which it was feared the Fund indirectly proposed to impose upon hospitals by their action in regard to the salaries of chaplains, came up. He then pointed out that he was quite confident that the Fund had never in any way done anything opposed to religious ministrations in hospitals, and the Bishop of Bristol in order that the point might be raised and disposed of at the next meeting of the Council had written to him asking that the Council might give expression to the opinion that nothing was further from their purpose than to do anything tending to a reduction of the means devoted to the supply of religious services in hospitals. He (the speaker) was perfectly sure that it was the wish and view of every member of the Council that what the Bishop of Bristol asked should be done.

The Chairman said that there was no intention on the part of the Fund to interfere with the religious ministrations to the sick, and the Bishop's proposal should be carried out.

